# Influence of linolenic acid in the production of jasmonate

**DOI:** 10.1186/1753-6561-8-S4-P178

**Published:** 2014-10-01

**Authors:** Vera Leite, Alexandre Santos, Murilo Innocentini, Geveraldo Maciel, Miriam Lourenço

**Affiliations:** 1 IFSULDEMINAS, Instituto Federal De Educação, Ciência e Tecnologia do Sul de Minas, Pouso Alegro, MG, Brazil; 2 UNAERP, Universidade de Ribeirao Preto, SP, Brazil

## Background

Research shows that jasmonic acid (JA) and Methyl Jasmonate (MJ), compounds that participate in the metabolism's control, development and protection of plant processes [1], also possess anticancer activities [2]. In studies, researchers found that Jasmonate were able to inhibit the growth of human cancer cells, and MJ induced death in lung carcinoma cells [3], breast, prostate, melanoma, lymphoma and leukemic cells [4], without reaching normal lymphocytes. The predominant route of obtaining these compounds is the vegetal extraction, however it takes about 800 kg of flowers of *Jasminum grandiflorum *to produce 1 kg of jasmone containing only 0.25% of AJ. However, the jasmonatos can also be produced by the microorganisms [5]. A microorganism which shows promising results in the production of Jasmonate is the fungus *Botryosphaeria rhodina*, making it possible to produce systems which can control the parameters involved in the fermentation process, including the use of precursors in this process.

## Methods

Inventories of line *Botryosphaeria rhodina *Kifn 3.1 were inoculated in petri dishes containing PDA culture medium, serving later to fermentation. The homogenate was inoculated in a 250 ml Erlenmeyer flask with 50 ml of media M_2. _The experiment was conducted using the methodology of 2^2 ^full factorial design, evaluating time ifluencia (7 and 14 days) and the addition of the precursor linolenic acid (0 and 1 µM). All experiments were performed in triplicate. After the period of each fermentation, the material was vacuum filtered and then subjected to extraction and analysis of the production of jasmonic acid. The extraction was by liquid-liquid partition using as extracting agent as ethyl acetate. The detection of the AJ extract was made by TLC (thin-layer chromatography comparative) Samples AJ standards (Sigma) were used as reference. Quantifying jasmonatos in extracts was performed by HPLC (High-performance liquid chromatography). The calculations of the factors influencing the time and addition of precursor and it's interaction were statistically analyzed by t-test at the 5% level of probability.

## Results and conclusions

Through the analysis of the CCDC fermentations with culture medium supplemented with linolenic acid was possible to verify that all samples had jasmonatos. The addition of linoleic acid (1 µM) induced inhibitory effect on the production of jasmonatos, however, the increase in time from 7 to 14 days to produce positively influences AJ. Thus it was concluded that the conditions evaluated is not necessary to supplement the culture medium with linoleic acid and increased time of fermentation favors the production of AJ by fungus *Botryosphaeriarhodina*.
